# Tacrolimus toxicity in islet transplantation due to interaction with macrolides

**DOI:** 10.1186/s40842-016-0019-7

**Published:** 2016-01-26

**Authors:** Kitty Kit-Ting Cheung, Peter Alexander Senior

**Affiliations:** 1 Department of Medicine and Therapeutics, The Chinese University of Hong Kong, Prince of Wales Hospital, New Territories, Hong Kong; 2 Department of Medicine, Clinical Islet Transplant Program, University of Alberta and Capital Health Authority, T6G 2C8 Edmonton, AB Canada

**Keywords:** Islet cell transplant, Type 1 diabetes, Tacrolimus, Clarithromycin, Adverse drug interaction

## Abstract

**Background:**

Drug interactions are an important risk in transplant patients. Case presentation: This case describes an incident where a patient with islet transplantation, who was using tacrolimus as part of the immunosuppressant regime, was started on a course of clarithromycin and experienced nephrotoxicity and neurotoxicity. He was an outpatient at that time and was managed with temporary cessation of tacrolimus until the tacrolimus level returned to target and his symptoms resolved. He recovered well and was resumed on his usual dosage of tacrolimus to prevent rejection of islets.

**Conclusion:**

Care should be taken with commonly used antibiotics to avoid potentially dangerous interactions with immunosuppressant drugs.

## Background

Solid organ transplant recipients are at risk for infections. Prompt antimicrobial therapy may be required. Physicians should be aware of the potentially dangerous interactions between some antibiotics and immunosuppressant drugs commonly used after solid organ transplant.

Tacrolimus is a potent immunosuppressant that is widely used for prevention of rejection in a variety of organ transplants. It is metabolized in the liver via cytochrome P450 (CYP) 3A4. Drugs which inhibit CYP3A4 can lead to elevated tacrolimus concentrations resulting in toxicity. We describe a case of a tacrolimus-clarithromycin interaction in an islet transplant recipient in order to highlight the significance of the interaction and to emphasize that alternatives were available to treat the patient should a similar situation recur.

## Case presentation

A 58 year old male who was diagnosed to have type 1 diabetes at age 11 had undergone successful islet transplantation due to life threatening hypoglycemia (pre-transplant Clarke score [1] = 7/7, HYPO score [2] = 1755 (severe > 1047), post-transplant Clarke score = 0/7, HYPO score = 0) 9 years earlier was maintained on tacrolimus (4.5 mg/day) and mycophenolate mofetil (2000 mg/day). Target levels for tacrolimus were 8–10 ng/ml. Prior to the transplant, the patient was on insulin 0.41 unit/Kg/day, and insulin was weaned off after the transplant.

In May 2015, he presented to the emergency department with a 3 day history of cough and wheeze without fever, sputum, or hemoptysis. At that time, his HbA1c was 6.1 %, urine albumin to creatinine ratio 5 mg/mmol, estimated glomerular filtration rate 70 mL min-1 per 1.73 m^2^, blood pressure 125/81 mmHg, body weight 61.1Kg, and body mass index 23.9 kg/m^2^.

A chest x-ray showed insignificant non-specific infiltrations. He was discharged with a 10 day supply of oral clarithromycin 500 mg twice daily and prednisone 50 mg once a day for treatment of suspected pneumonia. The patient asked both the physician and the dispensing pharmacist specifically whether there might be any interaction with his transplant medications and was assured there was none.

Five days later, the patient developed severe bilateral hand tremors and hyperglycemia. Clinical assessment in the transplant program confirmed significant involuntary bilateral fine hand tremors, marked hyperglycemia (22 mmol/l), but no fever and normal respiratory examination. Laboratory investigations confirmed extremely high concentrations of tacrolimus (47.2 ng/ml compared with 8.1 ng/ml prior to this incident) but fortunately only mild nephrotoxicity (serum creatinine 148 umol/l compared with 111 umol/l before).

Tacrolimus toxicity due to a drug interaction between tacrolimus and clarithromycin was the most likely cause for nephrotoxicity and neurotoxicity. Hyperglycemia was likely due to a mixture of tacrolimus toxicity and the side effect of prednisone. Tacrolimus toxicity could result in an acute decline in renal function which was observed in this case. Acute rejection could cause hyperglycemia but would not cause tremors.

Clarithromycin and prednisone were withheld pending confirmation of tacrolimus toxicity and assessment of renal function. Hyperglycemia was managed using subcutaneous insulin (0.13 unit/Kg/day) and cessation of the prednisone. In the absence of any signs or symptoms of ongoing infection, no antibiotics were given. In retrospect, while prednisone might have been warranted as one of the initial treatments in the emergency department due to wheeze, the duration (10 days) and dosage (50 mg) prescribed were excessive.

Tacrolimus levels and renal function were monitored twice weekly. By 3 days, the tacrolimus level was 13.3 ng/ml and the tremors had resolved. Tacrolimus was resumed at his usual dose and maintained in the target range (8–10 ng/ml).

Insulin therapy was effective to control the hyperglycemia and was gradually withdrawn over 2 weeks. Capillary blood glucose monitoring, off insulin, showed fasting glucose levels consistently around 7 mmol/l, suggesting adequate islet graft function. His renal function returned to baseline (creatinine 117 umol/L) at the same time.

## Conclusions

CYP 3A4 is the liver enzyme that metabolizes tacrolimus, whereas macrolide antibiotics, such as clarithromycin, have been reported to form complexes with CYP 3A4 and inhibit the metabolism of other drugs [3]. The use of both drugs together will result in increase in circulating tacrolimus concentrations, leading to an increased risk of adverse effects such as nephrotoxicity, neurotoxicity, and hyperglycemia [4, 5].

There are six cases in the literature involving tacrolimus-clarithromycin interactions. Wolter et al. reported the first case in which a renal transplant patient whose tacrolimus level increased from 9 to 29 ng/ml 3 days after the initiation of oral clarithromycin 500 mg twice daily for suspected pneumonia. On the sixth day of clarithromycin treatment, the tacrolimus concentration peaked at 36 ng/ml with a concurrent rise in serum creatinine from 327 umol/l to 422 umol/l. Tacrolimus was then withheld and the patient finished a two-week course of clarithromycin with normalization of the tacrolimus and creatinine levels [6]. Gomez et al. published two kidney transplant recipients who, after 9 doses of clarithromycin prescribed for respiratory infection, suffered from tacrolimus toxicity resulting in a concentration peaked at around 30 ng/ml and significant rise in serum creatinine in both cases. Clarithromycin was discontinued and tacrolimus was withheld for 1 dose, then restarted at 75 % of the original dose. The serum creatinine levels were back to baseline 10 days after the cessation of clarithromycin [7]. In a retrospective study of the nephrotoxicity of tacrolimus in 22 renal transplant patients, one patient was identified to have suffered from a rise in both the drug level and serum creatinine attributed to concomitant use of clarithromycin [8].

This adverse drug interaction is not limited to kidney transplant patients. Ibrahim et al. described this interaction in a bone marrow transplant recipient. The patient was on tacrolimus for the prevention of graft-versus-host disease after receiving an unrelated donor hematopoietic stem-cell transplant. He was treated with oral clarithromycin 500 mg twice daily as part of the antibiotic regime in an episode of acute lobar pneumonia 4 months after the procedure. Totally, 8 doses of clarithromycin 500 mg were given with tacrolimus 4 mg, resulting in an elevation of tacrolimus from 6.3 ng/ml to 10.1 ng/ml, and serum creatinine from 97 umol/l to 150 umol/l. Clarithromycin was stopped and tacrolimus was withheld. After 2 days of discontinuation of clarithromycin, tacrolimus was resumed at 3 mg twice daily. Unfortunately, the patient developed severe pneumonia and died shortly afterwards [9]. Similarly, such interaction was documented in a pharmacokinetic study on a heart transplant recipient who was on long-term tacrolimus for immunosuppression who required a seven-day course of clarithromycin as part of the triple therapy against *Helicobacter pylori* identified in gastroscopy. Tacrolimus was reduced from 6 to 4 mg per day pre-emptively before clarithromycin use and 3 doses of tacrolimus was withheld at the completion of the clarithromycin course, with pharmacokinetic parameters recorded along the line. The tacrolimus dose were then gradually increased over the next 4 days up to 3 mg per day on the seventh day from discontinuation of clarithromycin. The pharmacokinetic profile revealed elevation of tacrolimus concentrations when clarithromycin was in use. The serum creatinine levels for this patient were relatively stable, and this might be due to a different types of transplanted organ and/or individual kidney function, as compared to the other 5 reported cases and our case in which serum creatinine levels were raised when the tacrolimus levels were high [10]. Though all of the previously reported cases focused on nephrotoxicity, neurotoxicity is another well documented adverse effect from tacrolimus overdose. Mechanisms underlying tacrolimus neurotoxicity include damage of the astrocytes at the blood–brain barrier, excess production of endothelin resulting in vasospasm causing local ischemia and edema at cerebrum, and alterations in mitochondrial function leading to apoptotic cell death [11].

In our experience, azithromycin, another macrolide antibiotic, is an alternative to clarithromycin because this antibiotic has a very similar microbiological spectrum while it does not interact significantly with CYP 3A4 enzyme. It should also be noted that other drugs which are metabolized via the same enzyme system should also be used with caution for patients on tacrolimus, such as: ketoconazole, itraconazole, troleandomycin, nelfinavir, ritonavir, and nefazodone [12].

To our knowledge, this case is the first case report of a tacrolimus-clarithromycin interaction in an islet transplant recipient. Clinicians need to be reminded of this significant drug interaction, which might occur within 3–5 days of concomitant administration, and consider other alternative antibiotics in treatment of infections.

### Consent

We confirm that the patient has given their consent for the Case reports to be published.

## Abbreviations

Cytochrome: CYP
